# Professional Identity Formation in medical school: One measure reflects changes during pre-clerkship training

**DOI:** 10.15694/mep.2018.0000041.1

**Published:** 2018-02-21

**Authors:** Adina Kalet, Lynn Buckvar-Keltz, Verna Monson, Victoria Harnik, Steven Hubbard, Ruth Crowe, Tavinder K. Ark, Hyuksoon S. Song, Linda Tewksbury, Sandra Yingling

**Affiliations:** 1 New York University School of Medicine; 2 Monson Consulting and Evaluation; 3 The New School; 4 University of British Columbia; 5 Georgian Court University; 6 University of Illinois College of Medicine

**Keywords:** Professional Identity Formation, Medical Students Professional Identity Essay, Professionalism, Assessment, Reflection

## Abstract

This article was migrated. The article was marked as recommended.

Professional Identity Formation (PIF), the process of internalizing a profession’s core values and beliefs, is an explicit goal of medical education. The Professional Identity Essay (PIE), a developmental measure of the extent to which individuals have a complex and self-defined understanding of their professional role, is a tool to both study and scaffold PIF. PIE staging has internal reliability and response process validity and correlates with a validated measure of moral reasoning. In this study, we investigate whether PIF, as measured by PIE, changes during pre-clerkship training.

Medical students in the class of 2019 completed the PIE during orientation to medical school (PIE#1) and 15 months later, during orientation to clerkships (PIE#2), to the same prompts. These written responses are PIF-staged by an expert rater.

On average, PIF scores reveal that 46% of the group remained at the same stage as they were on entry to medical school, 42% scored at a higher stage of PIF, and 15% of students scored at a lower stage of PIF after pre-clerkship training.

This result suggests that medical students are heterogeneous with respect to the development of their medical PIF early in medical school training.

## Introduction

Our understanding of medical professionalism is rapidly evolving while, at the same time, we debate how an individual’s professional identity and behavior is shaped ( Irby & Hamstra, 2016). A historically dominant view was that professional behavior springs from enduring and immutable qualities of an individual’s personality or character that were formed early in life, sometimes called virtues-based professionalism. This view has been largely replaced by the notion that professionalism is shaped gradually over time by external contexts, instruction, and reinforcement, and is manifested almost entirely by observable behaviors (Ginsburg and Stern 2004; Irby and Hamstra 2016). An emerging framework, Professional Identity Formation (PIF) focuses instead on professionalism as a reflection of an ongoing developmental process that is shaped by the beliefs and values of the individual as well as by the environment, including both the formal planned and informal “hidden” curricula of medical education, healthcare delivery, and larger social forces ( Hafferty & Levinson, 2008). This approach encompasses aspects of virtue-based professionalism and also acknowledges the time-tested observation that people frequently know the right thing to do and yet often fail to act on their knowledge or convictions ( Rest, Bebeau, & Thoma, 1999). The PIF approach is informed by decades of research in moral psychology on how to increase the consistency between knowing and behaving ( Rest, 1994). PIF refers to an increasingly internalized, and therefore self-defined, set of core values and beliefs about professional responsibilities that can be accessed to guide behavior, even in highly complex settings.

PIF’s theoretical foundation dates back over 50 years, informed by the constructive-developmental theories of morality of Jean Piaget, Lawrence Kohlberg, and James Rest ( Hafferty & Levinson, 2008), the adult developmental psychology models of mental complexity of Robert Kegan ( Kegan, 1995), and the ethics principles applied to professional education ( M. J. Bebeau, 2002). The central premise of constructive-developmental theory is that our ability to actively create moral meaning grows in stages that represent increasing complexity of thought ( Rest, 1994). Over the course of higher education, students can become increasingly capable of making sense of demanding or ambiguous problems; this ability is essential for maximizing effectiveness and consistent behavior in professional roles that will meet the expectations of that profession ( Baxter Magolda, 2008; Baxter Magolda, King, Taylor, & Wakefield, 2012). Engaging students in complex experiences and guiding them to reflect on and make sense of these experiences facilitates the increasing internalization of a more complex and increasingly self-defined and regulated identity ( Sharpless et al., 2015; Wald, 2015).

The Carnegie Foundation ( Cooke, Irby, & O’Brien, 2010) calls for an evidence-based approach to understanding the development of an ethical medical professional identity. Leading authorities on teaching and assessing professionalism define professional identity as a “.. representation of self, achieved in stages over time during which the characteristics, values, and norms of the medical profession are internalized,” ( Cruess, Cruess, Boudreau, Snell, & Steinert, 2014), and note that more research is needed to guide medical education practice and scholarship.

Interest in medical PIF is growing. Prior to the 2010 publication of the Carnegie Report Educating Physicians: a Call for Reform of Medical School and Residency, ( Cooke et al., 2010) medical sociologists challenged medical education to move away from the simplistic regulatory view that professionalism is a list of traits and behaviors to be upheld. Rather, they argued, professionalism is a complex construct influenced by the expectations of society and expressed as a social contract, and the consequent privileges and responsibilities that professionals have to society is often manifested behaviorally ( Hafferty & Levinson, 2008; Morrison, Dowie, Cotton, & Goldie, 2009). Papadakis et al. (2005) found that lapses in the professional behavior of practicing physicians can be predicted by unprofessional behaviors, such as irresponsibility or a diminished capacity for self-improvement, as medical students. Educators hearkened to this evidence and redoubled efforts to address PIF early in training ( Rabow, Remen, Parmelee, & Inui, 2010; Wald, 2015), seeking to understand unprofessional behavior as observable manifestations of incomplete or disrupted PIF ( Holden, Buck, Clark, Szauter, & Trumble, 2012). The PIF framework then became a useful guide with which to design instructional activities for students. In an effort to support a healthy PIF, structured reflections and simulations as well as systems level actions were implemented to sustain the focus of medical practice on excellent patient care ( Branch et al., 2017; Lesser et al., 2010; Wald et al., 2015).

### Measuring PIF

Measures of PIF within professional education developed by Bebeau ( M. J. Bebeau & Monson, 2008) in the 1990s focus on survey-type tools such as the Professional Role Orientation Inventory ( M. J. Bebeau, Born, & Ozar, 1993). More recently, Bebeau and Lewis ( M. Bebeau & Lewis, 2003) developed an essay assessment of PIF based on Kegan’s Subject-Object Interview (SOI) in their work with professional military cadets. The SOI is an in-depth interview-based assessment of adult identity development defined as stages of cognitive, emotional, and social capacities that progress from less to more complex across the lifespan; extensive training in the interview and coding protocol is required. In validation studies using the Washington University Completion Test and the SOI, Kegan and colleagues estimate that 58 percent of adults fail to achieve self-authorship, characterized by greater effectiveness in managing self and others ( Kegan, 1995). This approach was adapted for professional education in the fields of dentistry, law, counseling, and medicine, and is now referred to as the Professional Identity Essay (PIE) ( M. J. Bebeau & Faber-Langendoen, 2014; M. J. Bebeau & Monson, 2012). The figure summarizes some key characteristics of individuals in each stage and in the transitions between stages. The PIE has primarily been used to provide formative feedback in order to promote and engage students in self-reflection and assessment. PIE studies in professional education yielded evidence of convergent construct validity examining correlations between SOI and PIE scores ( V. E. Monson & Hamilton, 2010). Additional construct validity of PIE is demonstrated by correlations between the Defining Issues Test 2 and PIE scores ( V. Monson, Roehrich, & Bebeau, 2008). The Defining Issues Test 2 is an extensively studied measure of the capacity for moral reasoning based largely on the same developmental theoretical frameworks as the PIE (e.g. the work of Piaget, Kohlberg, Kegan, Rest) ( Rest et al., 1999). We have previously reported that PIE stage scores from entering medical students 1) distributed similarly to those of age-matched students in other professions, and 2) students found these scores meaningful, suggesting there could be utility in measuring PIF scores in medical education ( A. Kalet et al., 2017).

There are no studies that evaluate PIF as measured by the PIE at different timesin an individual medical student’s training. However, a significant body of research from dental education shows that moral capacities can be supported by a focused ethics and professional curriculum. Law education research has demonstrated differences in PIF between entering students, early career graduates, and practicing lawyers considered exemplars in the field ( Monson VE and Hamilton 2010). Because a method to study PIF over time would be of great interest to the medical education community, we are conducting a longitudinal study of PIF among medical students in one school in the United States. We hypothesize that PIE stage scores will progress over time in medical training and we anticipate that patterns of progress will vary between students. In this paper, we report early study results.

**Figure 1.**
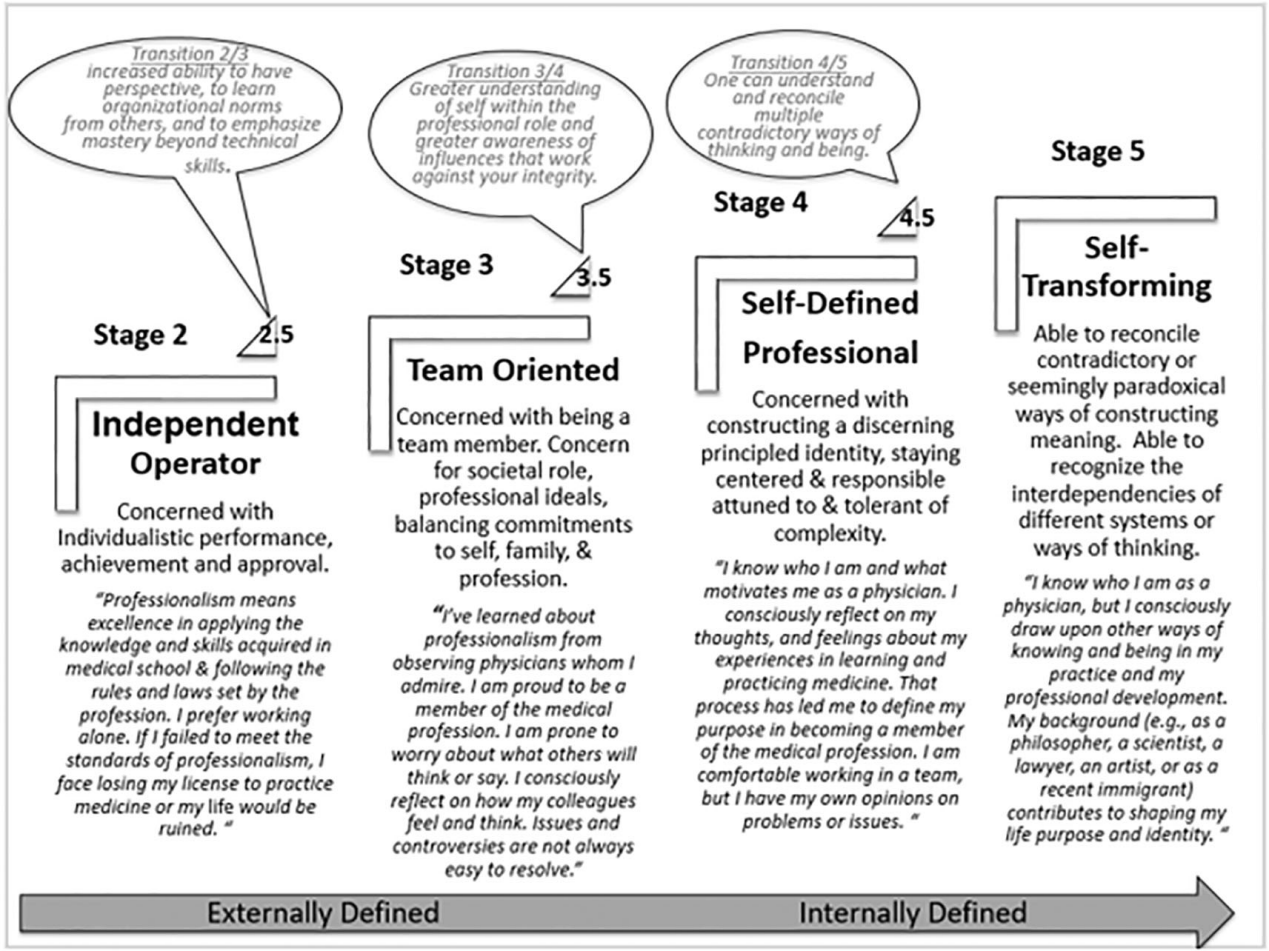
A framework of Medical Professional Identity Formation based on Bebeau’s adaptation of Kegan’s Stages of Mental Complexity, measured by the Professional Identity Essay (PIE) modified for the Professions. ( M. J. Bebeau &amp; Monson, 2008). Adults spend most of their lives in between the full stages in the transition from external to internal definition. Stage 1 pertains to infancy and childhood. A full Stage 5 exists primarily in theory. Half stages represent transitions between stages and are assigned by individuals trained in the use of coding criteria for professional identity developed by Bebeau and Lewis ( M. Bebeau &amp; Lewis, 2003). and guided by the Subject-Object Interview (SOI). ( M. J. Bebeau et al., 1993). Also depicted are descriptive labels proposed for the stages based on key characteristics of each stage and exemplar quotes for each full stage.

## Methods

### Curricular Context

The NYU School of Medicine has a four-stage curriculum with 1) a 15-month pre-clerkship training (Curriculum Stage 1), 2) a 12-month core clerkship rotation (Curriculum Stage 2), 3) a six-month individualized exploration phase where students engage in elective/selective experiences and prepare for and take both a school-based comprehensive clinical skills exam and the USMLE Step 1 (Curriculum Stage 3), and 4) an 11-month career preparation phase during which students complete advanced clinical clerkships, a scholarly concentration, electives, and apply for graduate medical education training slots (Curriculum Stage 4). Similar to many LCME-accredited medical schools, we have developed a rich medical professionalism curriculum that is integrated into core clinical skills training and includes case-based and student-led experiences ( Horlick, Masterton, & Kalet, 2009; King, Baxter Magolda, Barber, Brown, & Lindsay, 2009; 2003; Rockfeld, Horlick, & Kalet, 2003). We have also developed an electronic student academic portfolio, a section of which is devoted to the promotion of learners’ professional development and includes six periodic reflections distributed over the four years ( Adina Kalet & Pusic, 2014; A. L. Kalet et al., 2007). Students receive written feedback on their reflections and may choose to review these reflections with their faculty advisor during regularly scheduled individual meetings. The PIE was integrated into this professionalism curriculum and formative portfolio review process.

### Procedures

The Professional Identity Essay (PIE) adapted for medical education elicits the respondent’s conceptualization of his or her professional role in society and measures the stages of mental complexity (i.e., psycho-social-emotional capacities) based on the responses. The narrative responses to nine prompts described below serve as the basis to assign a PIF stage to the individual student’s document.

#### Writing Baseline PIE (PIE #1)

During the week of medical school orientation (August 2015), entering medical students (graduating class of 2019) attended a 30-minute large group introduction to the concept of professional identity formation based on the framework proposed by Cruess ( Cruess et al., 2014) and its developmental nature as conceptualized by Bebeau (M. J. Bebeau & Faber-Langendoen, 2014). Following this presentation, the students were given three days to submit the PIE electronically, which took an average of one hour to complete. Students were told the purpose of completing the PIE was to engage them in self-assessment, reflection, and goal setting, and students were encouraged to respond as fully as possible to each of the questions. The prompts were as follows:

• What does being a member of the medical profession mean to you? How did you come to this understanding?
• What do you expect of yourself as you work towards becoming a full-fledged physician?
• What will the profession expect of you?
• What conflicts do you experience or expect to experience between your responsibility to yourself and others-patients, family, and profession? How do you resolve them?
• What would be the worst thing for you if you failed to live up to the expectations you have set for yourself?
• What would be the worst thing for you if you failed to live up to the expectations of your patients?
• What would be the worst thing for you if you failed to live up to what society expects of physicians? How did you come to this understanding?
• Think of a physician you consider an exemplar of professionalism. Describe why you chose this person, illustrating with an incident or pattern of decisions or actions that supports your choice.
• Reflect on your experiences in medical school or in the community that have been critical in fostering change in your understanding of what it means to be a professional - to be a physician.

#### The PIE Feedback Report

The de-identified responses were sent to Dr. Verna Monson for scoring (see below.) Once scoring was complete, a feedback report was prepared for each student that included a report of the score as well as a description of the Professional Identity Formation (PIF) stages, the prompts, and the student’s responses, along with individualized narrative feedback explaining why their essays where assigned the particular stage (an example of this report has been previously published) (A. Kalet et al., 2017).

#### Debriefing and Reflecting on the Baseline PIE (PIE #1)

After receiving these reports, students gathered in a large group session for a brief didactic review of the theory and development of professional identity formation (10 min) and were asked to write reflections in their e-portfolios (while still in the large group setting) to the following prompts: ‘Now that you have received feedback on your PIE, write your first impressions. What surprised you? What “rings true”? What information is helpful to you?’ Each student was also asked to: ‘Write to your future self.. What do you want to remind yourself about - your concerns and aspirations as a beginning medical student? What challenges do you anticipate as you begin to act and feel like a physician?”

#### Writing and Debriefing the Follow-up PIE at 18 months (PIE #2)

At the end of pre-clerkship training (month 15 of Curriculum Stage 1), the Class of 2019 repeated the exercise of responding to the same nine prompts using the same digital format. PIEs were stage-scored and three months later students received their PIE Feedback Report containing the PIF Stage score, individualized feedback, their baseline (previous) PIF Stage, as well as the ‘note to future self’ they had written during the first month of medical school.

A debriefing session, similar to the first, briefly reviewed the concepts of PIF (10 min) and the students again submitted reflections to their e-portfolios (30 min was allotted in the session, but students were free to supplement their writing afterward). The session was concluded with a large group discussion about student experiences of PIF and how professional development might progress during their first three months of core clerkships; the Deans for Student Affairs (LBK, LT) and Curriculum (VH), the Director of Integrated Clinical Skills (RC) and Lead Professionalism Faculty (AK) were participants.

## Scoring and Data Analysis

De-identified text data from consenting students was analyzed as permitted by our IRB-approved Medical Student Research Registry, protocol number IRB #i08-674 ( Gillespie et al., 2016). In this study PIE data from 128/130 consenting students was available for analysis and student tracking.

### Professional Identity Essay scoring

The PIEs were all scored by VM, an adult developmental educational psychologist with extensive experience and expertise in measurement of moral capacities in professional education, and in particular using this tool ( M. J. Bebeau & Monson, 2012; Lahey, Souvaine, Kegan, Goodman, & Felix, 1988) (Previously reported29 Inter-rater ICC .83, 95% CI [.57 - .96], intra-rater ICC .85, 95% CI [.50 - .93]). While she has no contact with the individual students, she was not blinded to whether she was scoring PIE #1 or PIE #2, which occurred at different times of the year.

#### Quantitative

We analyzed de-identified data from consenting students with complete data (N=122 of 128 students). PIF stages were categorized by stages and half-stages (transitional, refer to figure for details). As the data is categorical, we conducted a Spearman’s Correlation coefficient between PIE 1 and PIE 2 and assessed the change in PIF for the group using a Chi Square analysis.

## Results

Student PIE #1 and PIE #2 stage scores were correlated (polychoric correlation= 0.460, p = 0.04). In aggregate, the scores shifted toward more mature stages (X2(9)=39.42, p<0.01, Table 1). This included 51 (42%) students whose PIF stage advanced: 38 by 0.5 stages (stage 3 to 3.5, 3.5 to 4), and 13 by a full stage. Fifty-six (46%) students remained at the same PIF stage and 14 (15%) students’ PIF stage was lower by 0.5 (1 score dropped a full stage from 3.5 to 2.5). Post-hoc chi square test found a significant number of individuals increased between stage 3.5 to 4 (p<0.01), and 3 to 4 (p<0.01). No significant differences were found in the number of individual student’s change between stage 3 to 3.5, or 3.5 to 4 and 2.5 to 3.0, and 2.5 to 3.5 (p>0.05).

**Table 1.**
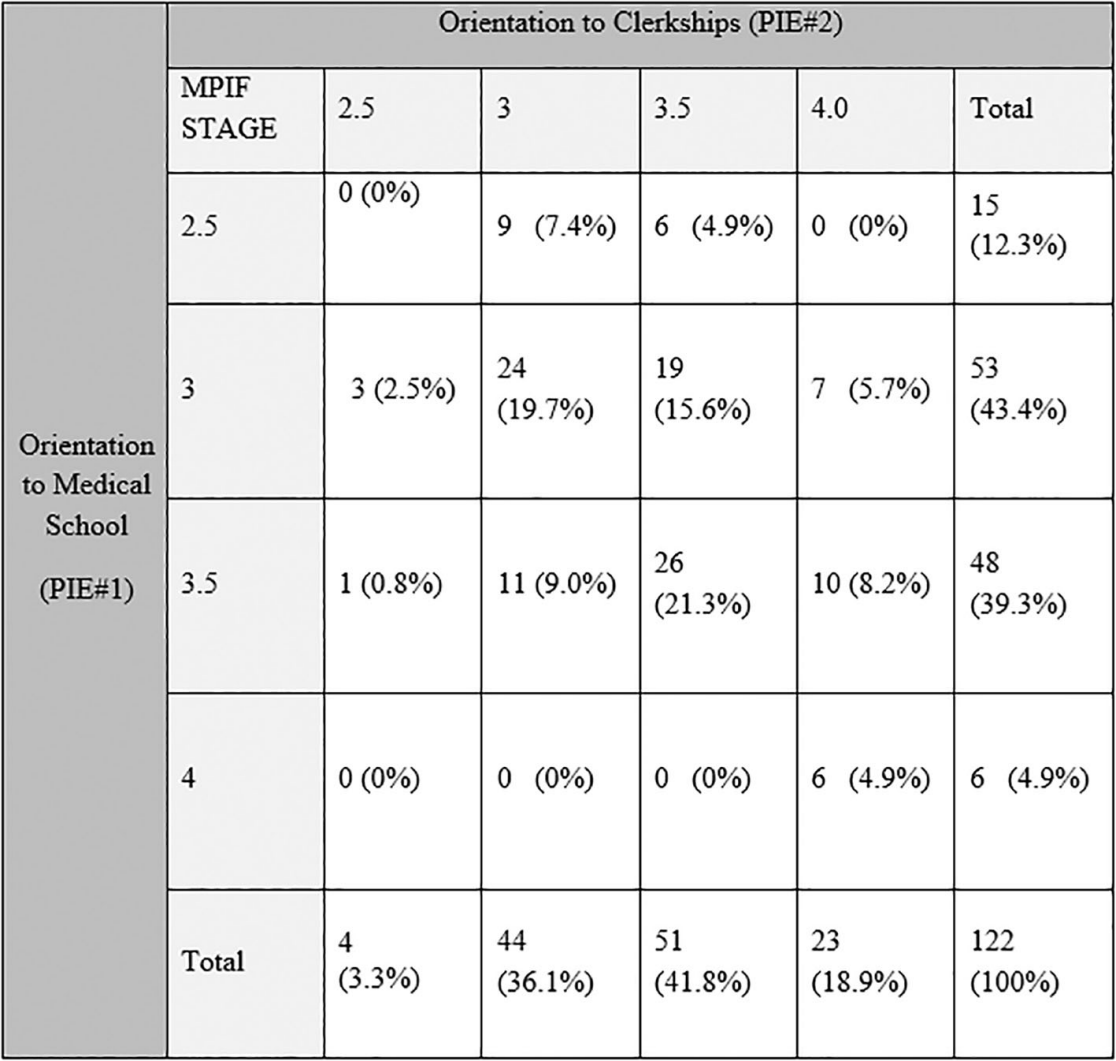
Crosstab of Individual Professional Identity scores at two times: Orientation to Medical school (PIE# 1) and Orientation to Clerkships (PIE# 2) 15 months later

## Discussion

Overall, for one cohort of medical students, PIE-based PIF stage scores increase over the first 15 months of medical school. Over time, we may be able to glean the meaning of individual variations in the PIF trajectory. As expected, on entry to medical school there was a range in student PIF scores similar to that seen in early stage professional students in general. We do not yet know enough about what influences PIF in medical school or when development is greatest or most vulnerable. However, theory suggests that developing self-authorship, a proposed surrogate marker for an ethical PIF, accelerates to maximal rate after college, when most individuals begin to juggle adult commitments and develop internal meaning-making that requires more complex thought ( Baxter Magolda, 2008; Baxter Magolda et al., 2012). Our baseline PIF stage data (at orientation to medical school) support previous findings that entering professional students tend to have higher stage PIF than the age-matched general population.

Although it will need to be confirmed, our data support the hypothesis that the PIE can be used to track the development of PIF throughout medical training (and beyond). This finding may allow us to learn more about the specific factors that shape and promote PIF and gain insight into what we might do to mitigate impeding factors. Others have described and evaluated strategies designed to promote PIF, conceptualized as an active constructive process of developing a mature and ethical identity as a physician in parallel to gaining a wide range of competencies critical to the role ( M. J. Bebeau & Monson, 2012). These strategies include, among others: reflective writing, mindfulness-based resilience training in response to challenging situations, and the use of eportfolios to cultivate PIF through integrating multiple learning opportunities and scaffolding mentoring relationships ( Wald et al., 2015). A measure of PIF, based on well-established developmental constructive theories and analysis of individual reflective comments on the PIE reports, may provide insights into influences and influencers or, in the lexicon of adult learning, critical incidents ( Metzirow, 1990). These insights would enable the community to conduct research clarifying how and why these curricular strategies work and therefore provide the evidence base to guide medical education practice and policy ( Cook, Bordage, & Schmidt, 2008; Rabow et al., 2010).

While it is reassuring that, in general, PIF stage advanced-suggesting that engagement in medical education moves many students from an externalized source of their PIF toward one that is internalized-individual patterns vary, including a subset of students who scored at a lower stage PIF 15 months into medical school. While this result may be ascribed to lack of precision in the measure, regression to the mean, or episodic lack of student effort, it may also indicate a meaningful regression in PIF stage. There is no reason to assume that the development of PIF is a smooth and linear process. It is more likely that it advances in fits and starts in response to developmental challenges. Developmental theory in moral psychology proposes that identity ( Rest et al., 1999) is relatively stable for long periods of time, punctuated by more dynamic “transitions” (represented by the PIF half stages) where it becomes less stable. It is also likely that the arc of PIF is a long one and, therefore, measurable growth in PIF might not be expected in every student after only one and a half years of training. For medical students, the most rapid increases in this development might be expected after pre-clinical training, in the early phases of clerkship rotations, and internship, where learners experience the predictable daily emotional and physical challenges of direct responsibility for clinical decision-making and patient care. Hopefully by following this cohort forward we can shed light on these issues.

In its current formulation, there is no concern about a ceiling effect in using PIE to measure PIF in medical trainees. While PIE stages 5 or greater are possible, they are not generally seen among groups of trainees and are uncommon among adults in general ( Kegan, 1995). Anecdotally, our students are requesting to understand “what a PIF Stage 5 looks like.” In response to this request, we hope to describe such individual exemplars as has been done in other fields ( Colby & Damon, 2014; Rule & Bebeau, 2005).

## Future Directions

The results of this study provide additional validity evidence for using the PIE as a measure of PIF in medical students in the context of formative feedback and reflection. Previously, we demonstrated 1) evidence for internal structure (inter- and intra-rater reliability of the staging of the PIE), 2) a positive and significant relationship to the Defining Issues Test 2, a validated measure of moral reasoning which is a theoretically related variable, and 3) evidence that students completing these essays and reflecting on the feedback from them understood the construct of PIF underlying this framework-suggesting response process validity evidence as described in the Messick validity model ( Cook & Beckman, 2006; A. Kalet et al., 2017). That PIF as measured by the PIE increases over time in some students and not others may enable us to understand the key influences that shape identity formation and therefore afford us an opportunity to design curriculum to promote PIF. Correlating PIE scores with academic performance or behavioral lapses in professionalism in medical school may ultimately guide remediation. Finally, an ability to predict serious problems in a given student at an early stage of training could prevent the longer-term consequences of poor performance for students, educators, and patients.

We cannot yet justify using the PIE stage score for high stakes decision-making such as promotion, remediation or dismissal. Understanding that measurement validity is increased with the use of multiple measures, multiple methods, and administered over time, we plan to administer the PIE a third time to these students later in medical training (American Educational Research Association, American Psychological Association, National Council on Measurement in Education, & Joint Committee on Standards for Educational and Psychological Testing, 2014). In the meantime, based on our experience, we do recommend the use of the PIE as a formative tool to facilitate a healthy, positive PIF and plan to re-administer the exercise to the Class of 2019 at the end of their acting internships. This “third point on the curve” will enable us to explore PIF as a developmental and predictive construct of both individual and group growth curves and as a complement to routinely collected behavioral measures of professionalism (and lapses thereof).

Our single school study findings have limited generalizability. The PIE is an essay-based measurement, which requires significant time and expertise to accurately score for stage and provide narrative feedback for reflection. However, given the lack of alternative validated and efficient measures of this construct, we view this as a tradeoff worth making because it will guide our next research efforts. Information gleaned from these studies may inform measures that are more streamlined.

Finally, we have found that providing numbered developmental stage information to students is controversial, especially for certain medical students who may be more performance-oriented than mastery goal-oriented when it comes to their own PIF ( Cutrer et al., 2017; Teunissen et al., 2009). In our prior work, we identified that students who embrace that they are novices are more likely to have positive responses to the curricular use of the PIE than those who do not articulate that they are early learners in a long process of PIF (A. Kalet et al., 2017). We are experimenting with using descriptive labels for the stages rather than numbers to shift focus away from numbers and instead stimulate curiosity among trainees about the PIF process and minimize negative or unhelpful aspects of the drive toward ‘high scores’ as achievement.

## Conclusion

In conclusion, this study supports the validity and meaningfulness of using the PIE in studying PIF. This is a complex area of investigation; entering medical students are heterogeneous with respect to their PIF score and its change over time during pre-clerkship training. Continued research is needed using the PIE as a measure of the effectiveness of a PIF curriculum and in exploring the nature of the development of students’ PIF and related psycho-social-emotional capacities.

## Take Home Messages

• The theory-grounded essay-based measure of PIF used in this study offers valid and meaningful insight into the development of PIF.
• PIF scores suggest that the development of medical PIF early in medical school training is not homogenous across medical students.
• Further research is needed using the PIE to explore the development of PIF within a professionalism curriculum.

## Notes On Contributors

Adina Kalet, MD, MPH directs the Research on Medical Education Outcomes unit at NYU School of Medicine, received the SGIM National Award for Scholarship in Medical Education in 2008, and is coeditor of the book *Remediation in Medical Education: A Midcourse Correction*, published by Springer.

Lynn Buckvar-Keltz, MD is the Director of the Medical Student Advisory Program at NYU School of Medicine and was Dean for Student Affairs for nine years. Her research interests include medical student professional development and preparedness for transition to residency.

Victoria Harnik, PhD is the Associate Dean for Curriculum at NYU School of Medicine, the content director for Anatomy in interdisciplinary pre-clerkship modules and co-authored Elsevier’s Integrated Anatomy and Embryology textbook and the iBook series, Virtual Prosections: The Series.

Verna Monson, PhD is a lifespan developmental psychologist in independent practice who received her doctorate from the University of Minnesota. Dr. Monson is an expert in assessing professional identity formation for graduate medical education and in remediating professionalism lapses. She is currently a consultant to NYU School of Medicine.

Ruth Crowe, M.D., PhD is the Director of Practice of Medicine, the core clinical skills training module given throughout pre-clerkship training at NYU School of Medicine. She is also Director of Integrated Clinical Skills, collaborating across the spectrum of undergraduate medical education to integrate core clinical skills training and assessment.

Hyuksoon S. Song, PhD instructs pre-service and in-service teachers in Georgian Court University, New Jersey as an assistant professor of education. He designs studies, analyzes data, and drafts manuscripts in medical education research.

Sandra Yingling, PhD is the Associate Dean for Educational Planning at the University of Illinois College of Medicine, and Assistant Professor in the Department of Medical Education. While at NYU, Dr. Yingling focused on evaluation and assessment, technology-enhanced education, and graduation requirements for clinical skills competence.

Tavinder K. Ark, PhD received her doctorate from the University of British Columbia in Measurement, Evaluation, and Research Methodology. Her research focuses on conducting simulations to better understand the impact of categorical data in statistical theories such as measurement invariance and generalizability theory.

Steven Hubbard, Ph.D. is studying data science and visualization at Parsons School of Design. He teaches courses at NYU in learning assessment, leadership, and career advising. Most recently, he conducted a research project with NYU’s Faculty Resource Network. His current research at Parsons concentrates on learning analytics and data visualization.

Linda Tewksbury, MD, MHPE is the Associate Dean of Student Affairs at NYU School of Medicine and the past Pediatric Clerkship Director. Dr. Tewksbury currently conducts research in the development of clinical reasoning in medical training.

## References

[ref1] American Educational Research Association, American Psychological Association, National Council on Measurement in Education, & Joint Committee on Standards for Educational and Psychological Testing . (2014). Standards for educational and psychological testing: American Educational Research Association.

[ref2] Baxter Magolda M. B. (2008). Three Elements of Self-Authorship. Journal of College Student Development. 49(4),269–284. 10.1353/csd.0.0016

[ref3] Baxter Magolda M. B. King P. M. Taylor K. B. & Wakefield K. M. (2012). Decreasing Authority Dependence During the First Year of College. Journal of College Student Development. 53(3),418–435. 10.1353/csd.2012.0040

[ref4] Bebeau M. & Lewis P. (2003). Manual for assessing and promoting identity formation.

[ref5] Bebeau M. J. (2002). The defining issues test and the four component model: contributions to professional education. J Moral Educ. 31(3),271–295. 10.1080/0305724022000008115 15027443

[ref6] Bebeau M. J. Born D. O. & Ozar D. T. (1993). The development of a professional role orientation inventory. Journal of the American College of Dentists. 60(2),27–33. 8408994

[ref7] Bebeau M. J. & Faber-Langendoen K. (2014). Remediating lapses in professionalism.In Kalet A. & Chou C. L. (Eds.), Remediation in medical education.(pp.103–127): Springer. 10.1007/978-1-4614-9025-8_7

[ref8] Bebeau M. J. & Monson V. E. (2008). Guided by theory, grounded in evidence: A way forward for professional ethics education.In Nucci L. & Narvaez D. (Eds.), Handbook of moral and character education.(pp.557–582). New York: Routledge.

[ref9] Bebeau M. J. & Monson V. E. (2012). Professional identity formation and transformation across the life span.In Mckee A. & Eraut M. (Eds.), Learning trajectories, innovation and identity for professional development.(pp.135–162). London: Springer. 10.1007/978-94-007-1724-4_7

[ref10] Branch W. T. Jr. Weil A. B. Gilligan M. C. Litzelman D. K. Hafler J. P. Plews-Ogan M. Rider E.A. Osterberg L.G. Dunne D. Derse A. R. Pittman J.R. Frankel R. M. (2017). How physicians draw satisfaction and overcome barriers in their practices: “It sustains me”. Patient Education and Counseling. 10.1016/j.pec.2017.06.004 28623052

[ref11] Colby A. & Damon W. (2014). Some do care. New York: Free Press.

[ref12] Cook D. A. & Beckman T. J. (2006). Current concepts in validity and reliability for psychometric instruments: theory and application. American Journal of Medicine. 119(2), 166.e167–116. 10.1016/j.amjmed.2005.10.036 16443422

[ref13] Cook D. A. Bordage G. & Schmidt H. G. (2008). Description, justification and clarification: a framework for classifying the purposes of research in medical education. Medical Education. 42(2),128–133. 10.1111/j.1365-2923.2007.02974.x 18194162

[ref14] Cooke M. Irby D. M. & O’Brien B. C. (2010). Educating physicians: a call for reform of medical school and residency.(Vol.16). San Francisco: John Wiley & Sons.

[ref15] Cruess R. L. Cruess S. R. Boudreau J. D. Snell L. & Steinert Y. (2014). Reframing medical education to support professional identity formation. Academic Medicine. 89(11),1446–1451. 10.1097/ACM.0000000000000427 25054423

[ref16] Cutrer W. B. Miller B. Pusic M. V. Mejicano G. Mangrulkar R. S. Gruppen L. D. Hawkins R.E. Skochelak S.E. Moore D. E. Jr. (2017). Fostering the Development of Master Adaptive Learners: A Conceptual Model to Guide Skill Acquisition in Medical Education. Academic Medicine. 92(1),70–75. 10.1097/ACM.0000000000001323 27532867

[ref17] Gillespie C. Zabar S. Altshuler L. Fox J. Pusic M. Xu J. & Kalet A. (2016). The Research on Medical Education Outcomes (ROMEO) Registry: Addressing Ethical and Practical Challenges of Using “Bigger,” Longitudinal Educational Data. Academic Medicine. 91(5),690–695. 10.1097/ACM.0000000000000920 26466377

[ref18] Hafferty F. W. & Levinson D. (2008). Moving beyond nostalgia and motives: towards a complexity science view of medical professionalism. Perspectives in Biology and Medicine. 51(4),599–615. 10.1353/pbm.0.0044 18997361

[ref19] Holden M. Buck E. Clark M. Szauter K. & Trumble J. (2012). Professional identity formation in medical education: the convergence of multiple domains. HEC Forum. 24(4),245–255. 10.1007/s10730-012-9197-6 23104548

[ref20] Horlick M. Masterton D. & Kalet A. (2009). Learning skills of professionalism: a student-led professionalism curriculum. Medical Education Online. 11. 28253791 10.3402/meo.v11i.4615

[ref21] Irby D. M. & Hamstra S. J. (2016). Parting the Clouds: Three Professionalism Frameworks in Medical Education. Academic Medicine. 91(12). 10.1097/ACM.0000000000001190 27119331

[ref22] Kalet A. Buckvar-Keltz L. Harnik V. Monson V. Hubbard S. Crowe R. Hyuksoon S. S. Yingling S. (2017). Measuring professional identity formation early in medical school. Medical Teacher. 39(3),255–261. 10.1080/0142159X.2017.1270437 28033728

[ref23] Kalet A. & Pusic M. V. (2014). Defining and Assessing Competence.In Kalet A. & Chou C. (Eds.), Remediation in Medical Education.(pp.3–15). New York: Springer. 10.1007/978-1-4614-9025-8_1

[ref24] Kalet A. L. Sanger J. Chase J. Keller A. Schwartz M. D. Fishman M. L. Garfall A.L. Kitay A. (2007). Promoting professionalism through an online professional development portfolio: successes, joys, and frustrations. Academic Medicine. 82(11),1065–1072. 10.1097/ACM.0b013e31815762af 17971693

[ref25] Kegan R. (1995). In over our heads: The mental demands of modern life. Cambridge: Harvard University Press.

[ref26] King P. M. Baxter Magolda M. B. Barber J. P. Brown M. K. & Lindsay N. K. (2009). Developmentally Effective Experiences for Promoting Self-Authorship. Mind, Brain, and Education. 3(2),108–118. 10.1111/j.1751-228X.2009.01061.x

[ref27] Lahey L. Souvaine E. Kegan R. Goodman R. & Felix S. (1988). A guide to the subject-object interview: Its administration and interpretation. Cambridge, MA: Harvard University, Graduate School of Education, Laboratory of Human Development.

[ref28] Lesser C. S. Lucey C. R. Egener B. Braddock C. H. 3rd Linas S. L. & Levinson W. (2010). A behavioral and systems view of professionalism. JAMA. 304(24),2732–2737. 10.1001/jama.2010.1864 21177508

[ref29] Masterton D. Horlick M. & Jones C. (2003). Professionalism begins in medical school. Virtual Mentor. 5(12). 10.1001/virtualmentor.2003.5.12.fred1-0312 23267565

[ref30] Metzirow J. (1990). Fostering critical reflection in adulthood. San Francisco: Jossey-Bass.

[ref31] Monson V. Roehrich S. & Bebeau M. (2008). Developing civic capacity of professionals: A methodology for assessing identity. Paper presented at the Annual Conference of the American Educational Research Association. New York.

[ref32] Monson V. E. & Hamilton N. W. (2010). Entering Law Students’ Conceptions of an Ethical Professional Identity and the Role of the Lawyer in Society. J. Legal Prof. 35,385. 10.2139/ssrn.1581528

[ref33] Morrison J. Dowie A. Cotton P. & Goldie J. (2009). A medical education view on sociological perspectives on professionalism. Medical Education. 43(9),824–825. 10.1111/j.1365-2923.2009.03444.x 19709006

[ref34] Rabow M. W. Remen R. N. Parmelee D. X. & Inui T. S. (2010). Professional formation: extending medicine’s lineage of service into the next century. Academic Medicine. 85(2),310–317. 10.1097/ACM.0b013e3181c887f7 20107361

[ref35] Rest J. R. (1994). Moral development in the professions: Psychology and applied ethics. Hillsdale (NJ): Erlbaum.

[ref36] Rest J. R. Bebeau M. J. & Thoma S. J. (1999). Postconventional moral thinking: A neo-Kohlbergian approach. Mahwah: Erlbaum.

[ref37] Rockfeld J. Horlick M. & Kalet A. (2003). Setting our own standards: a student-led professionalism curriculum for preclerkship students. Medical Education. 37(5),483. 10.1046/j.1365-2923.2003.01502_12.x 12709207

[ref38] Rule J. T. & Bebeau M. J. (2005). Dentists who care: Inspiring stories of professional commitment. Chicago: Quintessence Publishing Company.

[ref39] Sharpless J. Baldwin N. Cook R. Kofman A. Morley-Fletcher A. Slotkin R. & Wald H. S. (2015). The becoming: students’ reflections on the process of professional identity formation in medical education. Academic Medicine. 90(6),713–717. 10.1097/ACM.0000000000000729 25881650

[ref40] Teunissen P. W. Stapel D. A. van der Vleuten C. Scherpbier A. Boor K. & Scheele F. (2009). Who wants feedback? An investigation of the variables influencing residents’ feedback-seeking behavior in relation to night shifts. Academic Medicine. 84(7),910–917. 10.1097/ACM.0b013e3181a858ad 19550188

[ref41] Wald H. S. (2015). Professional identity (trans)formation in medical education: reflection, relationship, resilience. Academic Medicine. 90(6),701–706. 10.1097/ACM.0000000000000731 25881651

[ref42] Wald H. S. Anthony D. Hutchinson T. A. Liben S. Smilovitch M. & Donato A. A. (2015). Professional identity formation in medical education for humanistic, resilient physicians: pedagogic strategies for bridging theory to practice. Academic Medicine. 90(6),753–760. 10.1097/ACM.0000000000000725 25901874

