# Supplementary material for: Professional Identity Formation in medical school: One measure reflects changes during pre-clerkship training
Source: MedEdPublish (2016). 2018 Feb 21;7:41. [Version 1] doi: 10.15694/mep.2018.0000041.1 (PMC10712001; doi:10.15694/mep.2018.0000041.1)
Supplement: Peer review history

# Reviewer report: Reviewer response for version 1

**DOI:** 10.21956/mep.19510.r28854

**Published:** 2019-05-04

**Authors:** Ken Masters

**Affiliations:** 1 Sultan Qaboos University

This review has been migrated. The reviewer awarded 3 stars out of 5 An interesting paper dealing with measuring and monitoring professional identity formation of medical students.The authors have explained in good detail the theoretical background, and then proceed to describe the use of the Professional Identity Essay (PIE) in this study.Overall, the study has been well-conducted, and the results are encouraging. I am, however, concerned at the reliance on one test only. A weakness of any assessment is the reliance on a single test, hence universities around the world use many systems – the PIE, just like any other system, can be gamed, and will be gamed if the students realise that the results will carry any weight in any kind of decision-making. This is particularly so, given that, after the first test, they have an in-depth discussion, realise how the 'thing works', and adapt their answers to it the next time it is run.Although the authors do note the correlation to the Defining Issues Test (not conducted in this case), they also point out that it is “based largely on the same developmental theoretical frameworks as the PIE” I think the authors could have explained the extent to which the similarity in the development of these two tests may automatically lead to similarity in scores – i.e. are they actually the same measurement, using the same theoretical framework, but just presented in a different way? This statement by the authors gives that impression, so the correlation is not surprising, and is also not any real triangulation. If this is not the case, however, then the authors should discuss this in a little more detail.I would be far more convinced if other tests, based on different theoretical frameworks, were found to have a high correlation with these results. That said, it is also true that practicalities force limits on the number of tests that can be run, but, we also know that a thing that ‘is not perfect but it’s the best we have’ can quickly becoming the defining metric in any system, and the imperfections forgotten.So, it is an interesting paper, but I think there should be far more caution in the suggestion that the PIE (or any other test) can be used by itself.

Reviewer Expertise:

NA

No decision status is available

# Reviewer report: Reviewer response for version 1

**DOI:** 10.21956/mep.19510.r28852

**Published:** 2018-05-24

**Authors:** Rachel Ellaway

**Affiliations:** 1 University of Calgary

This review has been migrated. The reviewer awarded 5 stars out of 5 Tracking and directing professional identity formation for trainee doctors is notoriously nebulous. Finding ways to add a degree of systematicity and rigour to this is to be applauded. However, I am left wondering whether there is a single one-size-fits-all linear path or goal in PIF. Like learning styles, using this as a formative and reflective mechanism rather than a summative or diagnostic measure is important, not least because assessment tends to drive gaming and resentment behaviours in those being assessed. To that end, the discussion and emerging issues are particularly important in articulating a future research agenda, both practically and conceptually, particularly in terms of the limits to which PIF can be captured in a quantified and linear fashion. It is good to see reflective work of this quality and I recommend this paper to those who are wrestling with these issues.

Reviewer Expertise:

NA

No decision status is available

# Reviewer report: Reviewer response for version 1

**DOI:** 10.21956/mep.19510.r28853

**Published:** 2018-02-22

**Authors:** Ian Wilson

**Affiliations:** 1 International Medical University

This review has been migrated. The reviewer awarded 4 stars out of 5 I have long argued that there is no instrument that can measure professional identity. Survey instruments I have looked at perform very poorly and do not correlate with each other.this paper represents a significant forward step and confirms that a qualitative approach provides much more information. I urge anyone interested in Professional Identity to read this paper

Reviewer Expertise:

NA

No decision status is available
